# Compliance with smoke-free legislation within public buildings: a cross-sectional study in Turkey

**DOI:** 10.2471/BLT.15.158238

**Published:** 2015-11-23

**Authors:** Ana Navas-Acien, Asli Çarkoğlu, Gül Ergör, Mutlu Hayran, Toker Ergüder, Bekir Kaplan, Jolie Susan, Hoda Magid, Jonathan Pollak, Joanna E Cohen

**Affiliations:** a Department of Environmental Health Sciences, Johns Hopkins Bloomberg School of Public Health, 615 N Wolfe Street (Office W7033B), Baltimore, MD 21205, United States of America (USA).; b Department of Psychology, Kadir Has University, Istanbul, Turkey.; c Izmir Dokuz Eylül School of Medicine, Izmir, Turkey.; d Hacettepe University Cancer Institute, Ankara, Turkey.; e World Health Organization Country Office, Çankaya, Ankara, Turkey.; f Ministry of Health General Directorate of Health Research, Ankara, Turkey.; g Institute for Global Tobacco Control, Johns Hopkins Bloomberg School of Public Health, Baltimore, USA.

## Abstract

**Objective:**

To investigate public compliance with legislation to prohibit smoking within public buildings and the extent of tobacco smoking in outdoor areas in Turkey.

**Methods:**

Using a standardized observation protocol, we determined whether smoking occurred and whether ashtrays, cigarette butts and/or no-smoking signs were present in a random selection of 884 public venues in 12 cities in Turkey. We visited indoor and outdoor locations in bars/nightclubs, cafes, government buildings, hospitals, restaurants, schools, shopping malls, traditional coffee houses and universities. We used logistic regression models to determine the association between the presence of ashtrays or the absence of no-smoking signs and the presence of individuals smoking or cigarette butts.

**Findings:**

Most venues had no-smoking signs (629/884). We observed at least one person smoking in 145 venues, most frequently observed in bars/nightclubs (63/79), hospital dining areas (18/79), traditional coffee houses (27/120) and government-building dining areas (5/23). For 538 venues, we observed outdoor smoking close to public buildings. The presence of ashtrays was positively associated with indoor smoking and cigarette butts, adjusted odds ratio, aOR: 315.9; 95% confidence interval, CI: 174.9–570.8 and aOR: 165.4; 95% CI: 98.0–279.1, respectively. No-smoking signs were negatively associated with the presence of cigarette butts, aOR: 0.5; 95% CI: 0.3–0.8.

**Conclusion:**

Additional efforts are needed to improve the implementation of legislation prohibiting smoking in indoor public areas in Turkey, especially in areas in which we frequently observed people smoking. Possible interventions include removing all ashtrays from public places and increasing the number of no-smoking signs.

## Résumé

**Objectif:**

Analyser le respect de la législation interdisant de fumer à l'intérieur des bâtiments publics ainsi que l'ampleur du tabagisme en extérieur en Turquie.

**Méthodes:**

Suivant un protocole d'observation standardisé, nous avons déterminé si des personnes avaient fumé et si des cendriers, des mégots de cigarettes et/ou des panneaux interdisant de fumer étaient présents dans 884 lieux publics sélectionnés au hasard dans 12 villes turques. Nous nous sommes rendus dans des bars/discothèques, cafés, bâtiments gouvernementaux, hôpitaux, restaurants, établissements scolaires, centres commerciaux, cafés traditionnels et universités où nous avons examiné les espaces intérieurs et extérieurs. Nous avons utilisé des modèles de régression logistique pour déterminer l'association entre la présence de cendriers ou l'absence de panneaux interdisant de fumer et la présence de personnes en train de fumer ou de mégots de cigarettes.

**Résultats:**

La plupart des lieux disposaient de panneaux interdisant de fumer (629/884). Nous avons observé au moins une personne en train de fumer dans 145 lieux, le plus souvent dans les bars/discothèques (63/79), les espaces-repas des hôpitaux (18/79), les cafés traditionnels (27/120) et les espaces-repas des bâtiments gouvernementaux (5/23). Dans 538 lieux, nous avons observé que des personnes fumaient à l'extérieur près de bâtiments publics. La présence de cendriers était positivement associée au fait de fumer à l'intérieur et à des mégots de cigarettes, rapport des cotes ajusté (RCa): 315,9; intervalle de confiance (IC) de 95%: 174,9–570,8 et RCa: 165,4; IC 95%: 98,0-279,1, respectivement. Les panneaux interdisant de fumer étaient négativement associés à la présence de mégots de cigarettes, RCa: 0,5; IC 95%: 0,3-0,8.

**Conclusion:**

Des efforts supplémentaires doivent être déployés afin d’améliorer l'application de la législation interdisant de fumer à l'intérieur des lieux publics en Turquie, en particulier dans les lieux où nous avons fréquemment observé des personnes qui fumaient. Les actions possibles pourraient consister à retirer tous les cendriers des lieux publics et à augmenter le nombre de panneaux interdisant de fumer.

## Resumen

**Objetivo:**

Investigar el cumplimiento público de la legislación que prohíbe fumar en edificios públicos y el grado de consumo de tabaco en las zonas exteriores en Turquía.

**Métodos:**

Mediante un protocolo de observación estandarizado, se determinó si se fumaba y si había ceniceros, colillas o señales de prohibido fumar en una selección aleatoria de 884 espacios públicos de 12 ciudades turcas. Se visitaron tanto espacios interiores como exteriores en bares/discotecas, cafeterías, edificios gubernamentales, hospitales, restaurantes, escuelas, centros comerciales, cafeterías tradicionales y universidades. Se utilizaron modelos de regresión logística para determinar la asociación entre la presencia de ceniceros o la ausencia de señales de prohibido fumar y la presencia de colillas o personas fumando.

**Resultados:**

La mayoría de espacios contaban con señales de prohibido fumar (629/884). Se observó al menos una persona fumando en 145 espacios, algo que se observó con más frecuencia en bares/discotecas (63/79), comedores de hospitales (18/79), cafeterías tradicionales (27/120) y comedores de edificios gubernamentales (5/23). En 538 espacios, se observó gente fumando en el exterior cerca de edificios públicos. La presencia de ceniceros se relacionó de forma positiva con el hecho de fumar en interiores y la presencia de colillas, cociente de posibilidades ajustado, CPa: 315,9; intervalo de confianza, IC, del 95%: 174,9–570,8 y CPa: 165,4; IC del 95%: 98,0-279,1, respectivamente. Las señales de prohibido fumar se relacionaron de forma negativa con la presencia de colillas, CPa: 0,5; IC del 95%: 0,3–0,8.

**Conclusión:**

Se necesitan esfuerzos adicionales para mejorar la aplicación de la legislación que prohíbe fumar en áreas públicas interiores en Turquía, especialmente en áreas en las que se han observado fumadores frecuentemente. Las posibles intervenciones incluyen eliminar todos los ceniceros de los lugares públicos y aumentar el número de señales de prohibido fumar.

## ملخص

**الغرض:**

التحقيق في درجة الامتثال العام للتشريعات المتعلقة بمنع التدخين داخل المباني العامة والوقوف على مدى انتشار تدخين التبغ في المناطق المفتوحة في تركيا.

**الطريقة:**

باستخدام بروتوكول المراقبة الموحد، قمنا بتحديد ما إذا كان تتم ممارسة التدخين، وما إذا كانت منافض السجائر، وأعقاب السجائر و/أو لافتات عدم التدخين موجودة في مجموعة مختارة عشوائيًا من 884 مكانًا عامًا في 12 مدينة تركية. وقمنا بزيارة أماكن داخلية مغلقة وخارجية في الحانات/النوادي الليلية، والمقاهي، والمباني الحكومية، والمستشفيات، والمطاعم، والمدارس، ومراكز التسوق، والمقاهي التقليدية، والجامعات. استخدمنا نماذج التحوف (Regression Model) اللوجيستي لتحديد العلاقة بين وجود منافض السجائر أو عدم وجود لافتات منع التدخين ووجود أفراد مدخنين أو أعقاب السجائر.

**النتائج:**

لم يكن لدى معظم الأماكن لافتات لمنع التدخين (بواقع 629 من بين 884 مكانًا). كما لاحظنا وجود شخص واحد على الأقل يقوم بالتدخين في 145 مكانًا، وهو الأمر الذي لوحظ في معظم الأحيان في الحانات/النوادي الليلية (63 من بين 79)، ومناطق تناول الطعام بالمستشفيات (18 من بين 79)، والمقاهي التقليدية (27 من بين 120)، ومناطق تناول الطعام في المباني الحكومية (5 من بين 23). أما بالنسبة لـ 538 مكانًا، فقد لاحظنا أن التدخين في الأماكن الخارجية يحدث بالقرب من المباني العامة. كان وجود منافض السجائر مرتبطًا ارتباطًا إيجابيًا بحدوث التدخين ووجود أعقاب السجائر في الأماكن الداخلية المغلقة، بنسبة احتمال معدّلة بلغت: 315.9؛ بنسبة أرجحية مقدارها ‏95%: 174.9–570.8 ونسبة الاحتمال المعدّلة: 165.4؛ ونسبة أرجحية مقدارها 95‏%: 98.0–279.1، على التوالي. وارتبطت لافتات منع التدخين ارتباطًا سلبيًا بوجود أعقاب السجائر، بنسبة احتمال معدّلة بلغت: 0.5؛ ونسبة أرجحية مقدارها ‏95%: 0.3–0.8.

**الاستنتاج:**

هناك حاجة إلى بذل جهود إضافية لتحسين تنفيذ التشريعات المانعة للتدخين في الأماكن العامة المغلقة في تركيا، وخاصة في المناطق التي غالبًا ما لاحظنا الأشخاص يقومون بالتدخين فيها. تشمل التدخلات الممكنة إزالة جميع منافض السجائر من الأماكن العامة وزيادة عدد لافتات عدم التدخين.

## 摘要

**目的:**

旨在调查土耳其公众遵守公共建筑内禁止吸烟法规的情况，并调查其在室外吸烟的情况。

**方法:**

通过采用标准化观察规程，并随机选取 12 个土耳其城市内 884 个公共场所进行观察，我们确定了是否吸烟与是否出现烟灰缸、烟头和/或是否张贴禁烟标志之间的联系。我们观察了酒吧/夜店、咖啡店、政府大楼、酒店、餐厅、学校、商场、传统咖啡屋和大学校园。并采用逻辑回归模型确定出现烟灰缸或没有禁烟标志，和出现抽烟者或出现烟头之间的联系。

**结果:**

大多数场所都有禁烟标志** **(629/884)。据观察，145 个场所内就有至少一人吸烟，吸烟情况多发现于酒吧/夜店** **(63/79)、酒店就餐区** **(18/79)、传统咖啡屋 ** **(27/120)** ** 和政府大楼就餐区** **(5/23)。我们还观察到， 538 个场所的户外吸烟区靠近公共建筑。烟灰缸的出现与室内吸烟和室内出现烟头正相关，调整后比值** **(aOR):315.9；95% 置信区间** **(CI)：174.9–570.8，调整后比值** **(aOR)：165.4；95% 置信区间** **(CI)：98.0–279.1。禁烟标志与出现烟头负相关，调整后比值** **(aOR)：0.5；95% 置信区间** **(CI)： 0.3–0.8.

**结论:**

需加大禁烟法规在土耳其室外公共区域（尤其是我们经常观察到有人吸烟的区域）的执行力度。可行干预包括清除所有公共场所内烟灰缸，并增加禁烟标志数量。

## Резюме

**Цель:**

Изучить соблюдение обществом законодательства, запрещающего курение в общественных зданиях, и выявить масштаб табакокурения на открытом воздухе в Турции.

**Методы:**

С помощью стандартизированного протокола наблюдения были определены случаи курения и наличие пепельниц, окурков или знаков, запрещающих курение, в выбранных случайным образом 884 общественных местах в 12 городах Турции. Были посещены внутренние и наружные помещения баров, ночных клубов, кафе, правительственных зданий, больниц, ресторанов, школ, торговых центров, традиционных кофеен и университетов. С помощью модели логистической регрессии была определена связь между наличием пепельниц или отсутствием знаков, запрещающих курение, и присутствием курящих лиц или наличием окурков.

**Результаты:**

В большинстве мест присутствовали знаки, запрещающие курение (629/884). В 145 местах наблюдения присутствовал как минимум один курящий человек. Чаще всего подобное встречалось в барах и ночных клубах (63/79), больничных столовых (18/79), традиционных кофейнях (27/120) и столовых правительственных зданий (5/23). В 538 местах наблюдалось курение на открытом воздухе вблизи общественных зданий. Наличие пепельниц было положительно связано с курением внутри помещений и наличием окурков, скорректированное отношение шансов, сОШ: 315,9; 95%-й доверительный интервал, ДИ: 174,9–570,8 и сОШ: 165,4; 95%-й ДИ: 98,0–279,1 соответственно. Наличие знаков, запрещающих курение, было отрицательно связано с наличием окурков, сОШ: 0,5; 95%-й ДИ: 0,3–0,8.

**Вывод:**

Необходимы дополнительные мероприятия для более эффективного исполнения законодательства, запрещающего курение внутри общественных зданий в Турции, особенно в местах, где курящие люди встречаются наиболее часто. В число возможных мероприятий входит удаление всех пепельниц из общественных мест и увеличение количества знаков, запрещающих курение.

## Introduction

To protect everyone from the detrimental effects of exposure to second-hand tobacco smoke,^1^^,^^2^ the World Health Organization’s Framework Convention on Tobacco Control has called for comprehensive legislation to eliminate tobacco smoking in all indoor public places and workplaces.^3^^,^^4^ In Turkey – ranked among the top 10 countries in the world for tobacco use in 2008^5^ – the mean cigarette consumption among the 41.5% of men and 13.1% of women who smoked was 20.3 and 15.3 per day respectively in 2012.^6^

Turkey passed a law in 2008 that prohibited smoking in indoor public places and workplaces.^7^ Cafes, restaurants, bars, nightclubs and other hospitality venues were given until July 2009 to comply with this legislation.^7^ Several studies have evaluated the impact of the legislation in eliminating smoking in public places in Turkey.^8^^–^^10^ Most were based on convenience sampling^10^ and on only a few types of public venues.^8^^–^^10^ The Global Adult Tobacco Survey has monitored trends in exposure to second-hand smoke in Turkey – based on self-reported exposure in health-care facilities, government buildings, transport hubs and some hospitality venues – but it does not verify if or where smoking is occurring in any of the reported locations.^6^^,^^11^ In an attempt to evaluate compliance with the legislation on smoking in indoor public places in Turkey more comprehensively, we adapted a guide on compliance studies that was published by the International Union Against Tuberculosis and Lung Disease, the Campaign for Tobacco Free Kids and the Johns Hopkins Bloomberg School of Public Health in 2014.^12^ We used the presence of individuals who were smoking and/or cigarette butts as indicators of non-compliance with the legislation and the presence of ashtrays, the absence of no-smoking signs and the presence of cigarettes for sale as possible facilitators of non-compliance. In addition to evaluating compliance with the legislation on indoor smoking, we assessed outdoor exposure to second-hand tobacco smoke near the buildings.

## Methods

### Study population

In this cross-sectional observational study, we studied public venues in one city in each of the twelve first-level subdivisions used in Turkey by the European Union’s Nomenclature of Territorial Units for Statistics: Aegean, north-eastern, middle, middle-eastern, south-eastern and western Anatolia, eastern and western Black Sea, Istanbul, eastern and western Marmara and Mediterranean. Our corresponding study cities were Adana, Ankara, Balikesir, Bursa, Erzurum, Gaziantep, Istanbul, Izmir, Kayseri, Samsun, Trabzon and Van respectively. Within the urban districts of each city, the Turkish Statistical Institute randomly selected either 10 sampling points for the three major cities (i.e. Ankara, Istanbul and Izmir) or five such points for the smaller cities. Around each sampling point, our fieldworkers visited the closest bars/nightclubs, cafes, government buildings, hospitals, restaurants, schools, shopping malls, traditional coffee houses and universities. The fieldworkers gradually expanded the search until one or two of each type of public venue had been located around each sampling point and a pre-specified target number of venues of each type had been located in each study city. The target numbers, which had been set by a consensus panel before the field work began (available from the corresponding author), took into account the size of the city, the rarity of the type of venue and the allocated fieldwork duration – of two weeks in each major city and one week in each smaller city. A letter from the Ministry of National Education authorized access to schools. All other venues allowed public access. The fieldwork was conducted in December 2012–January 2013 in Ankara, Istanbul and Izmir and in May–July 2013 in the rest of the study cities. Institutional review boards at the Johns Hopkins University in Baltimore (United States of America) and at Doğuş University in Istanbul (Turkey) approved the study protocol.

### Data collection

Following a standardized protocol, trained fieldworkers conducted all the observations working in pairs and visited each study venue during the venue’s regular working hours. In each visited venue, the fieldworkers followed a standard itinerary and evaluated a pre-specified number of study locations. In government buildings, hospitals, schools, shopping malls and universities, the locations included – when present – the main entrance, a corridor, a stairwell, a waiting room or common area, classrooms, offices that were open to the public, a toilet area near a dining area and a dining area. In hospitality venues, the fieldworkers entered the venue, sat as customers, visited the toilet area and observed the other areas available in the venue. Fieldworkers also observed the outdoor area near the main entrance as well as any gardens or patios that belonged to the venues. In each study location, the fieldworkers recorded the number of people present, the number of people smoking, the presence or absence of cigarette butts, cigarette sales, ashtrays and no-smoking signs, the visibility of any no-smoking signs – i.e. whether the fieldworkers considered such signs to be obvious or tucked away where few visitors would notice them – and whether the no-smoking signs they saw, if any, included information on fines for smoking in the venue. As the legislation on the prohibition of smoking in Turkey did not apply to outdoor areas, at the time of the fieldworkers’ visits, any sign posted at the entrance to a venue was assumed to apply to the venue’s indoor locations.

In each of a random subset of 72 bars/nightclubs, we used a SidePak AM510 personal aerosol monitor (TSI, Shoreview, USA) to measure air concentrations of particulate matter with a diameter of less than 2.5 μm (PM_2.5_). We measured for 5 minutes outside the venue – at least 10 m from the entrance – for 20 minutes in the main bar area, for 5 minutes on the patio or terrace if present and, finally, for 5 minutes outside the venue but near the entrance.^13^^,^^14^ For each sampling location, the number of people and smokers and the exact date and time that the air monitoring was started and finished were recorded.

### Data analysis

We determined the percentage of the visited venues of each main type in which at least one individual who was smoking, at least one ashtray, at least one cigarette butt and at least one no-smoking sign were observed in the indoor study locations and, separately, in the outdoor study locations. In addition to reporting overall percentages for all 12 study cities, we used Fisher’s exact test to compare percentages between the three larger study cities and the other, smaller study cities. For the non-hospitality venues – i.e. government buildings, hospitals, schools, shopping malls and universities – we used the same protocol to compare the observations made in dining areas with those made in non-dining areas.

We also investigated the association between each of three possible facilitators of non-compliance with the so-called smoke-free legislation – i.e. the presence of ashtrays, the absence of no-smoking signs and the presence of cigarette sales – and either the presence of at least one individual who was smoking – as a marker of current smoking – or the presence of at least one cigarette butt – as a marker of past smoking.^15^ For this, we used logistic regression models that were either unadjusted or adjusted for other characteristics that the fieldworkers recorded, including the type of location. Those models, which provided unadjusted odds ratios and adjusted odds ratios (aOR) with 95% confidence intervals (CI), used generalized estimating equations to take account of the clustering of study locations within study venues and the consequent lack of independence between most observations.^16^ Generalized estimating equations were not used for cigarette sales since these were only recorded at venue level. All analyses were performed using Stata version 13.1 (StataCorp. LP, College Station, USA).

## Results

### Venues observed

The fieldworkers’ observations, made in a total of 884 venues, covered 3661 indoor locations – in which 34 651 people were observed – and 1356 outdoor locations – in which 14 489 people were observed (Table 1). Indoor dining areas were observed in 244 of the non-hospitality study venues: 23 (17%) of the 135 government buildings, 79 (89%) of the 89 hospitals, 35 (67%) of the 52 malls, 73 (54%) of the 134 schools and 34 (92%) of the 37 universities.

**Table 1:** Observations on compliance with smoke-free legislation in 12 cities, Turkey, 2012–2013

Location, venue type	No. of venues	No. of locations	No. of people observed	Mean no. of smokers observed per venue	No. (%) of venues with observed:			
					Smoking	Ashtray(s)	Cigarette butt(s)	No-smoking sign(s)
**Indoors**	884	3 661	34 651	1.4	145 (16.4)	144 (16.3)	165 (18.7)	629 (71.2)
University^a^	37	262	1 816	0.5	1 (2.7)	3 (8.1)	4 (10.8)	25 (67.6)
School^a^	134	960	7 192	0.1	7 (5.2)	9 (6.7)	14 (10.4)	73 (54.5)
Government building^a^	135	660	4 972	0.3	8 (5.9)	9 (6.7)	11 (8.1)	98 (72.6)
Shopping mall^a^	52	273	5 187	0.6	4 (7.7)	3 (5.8)	9 (17.3)	44 (84.6)
Hospital^a^	89	513	7 297	1.2	19 (21.3)	19 (21.3)	24 (27.0)	66 (74.2)
Restaurant	171	393	2 789	0.8	12 (7.0)	11 (6.4)	10 (5.8)	124 (72.5)
Modern cafe	67	154	799	0.2	4 (6.0)	4 (6.0)	5 (7.5)	42 (62.7)
Traditional coffee house	120	180	2 004	1.5	27 (22.5)	23 (19.2)	25 (20.8)	103 (85.8)
Bar or nightclub	79	266	2 595	9.0	63 (79.7)	63 (79.7)	63 (79.7)	54 (68.4)
**Outdoors**	884	1 356	14 489	3.8	538 (60.9)	368 (41.6)	782 (88.5)	NR
University	37	77	1 329	5.6	26 (70.3)	23 (62.2)	32 (86.5)	NR
School	134	268	4 042	1.1	58 (43.3)	5 (3.7)	124 (92.5)	NR
Government building	135	148	721	1.6	76 (56.3)	43 (31.9)	118 (87.4)	NR
Shopping mall	52	113	1 515	8.6	40 (76.9)	32 (61.5)	47 (90.4)	NR
Hospital	89	156	3 199	10.6	77 (86.5)	57 (64.0)	88 (98.9)	NR
Restaurant	171	230	1 112	1.8	85 (49.7)	62 (36.3)	133 (77.8)	NR
Modern cafe	67	96	413	1.6	30 (44.8)	28 (41.8)	52 (77.6)	NR
Traditional coffee house	120	164	1 190	4.7	89 (74.2)	90 (75.0)	116 (96.7)	NR
Bar or nightclub	79	104	968	5.1	57 (72.2)	28 (35.4)	72 (91.1)	NR
**Indoors and outdoors**	1 768	5 017	49 140	2.6	683 (38.6)	512 (29.0)	947 (53.6)	NR

NR: not recorded.

^a ^These venues included dining and non-dining areas.

### Indoor locations

**Fig. 1:**
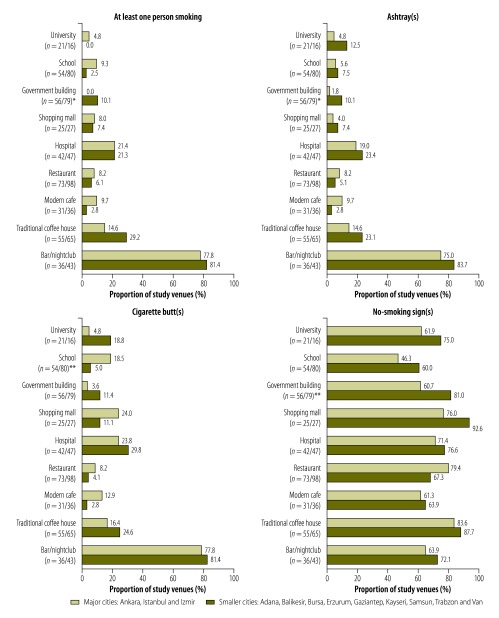
Indoor observations of smoking, ashtrays, cigarette butts and no-smoking signs in 12 cities, Turkey, 2012–2013

The presence of smoking, ashtrays and cigarette butts in indoor locations differed markedly by venue type (Table 1) but not study city size (Fig. 1).

* *P* ≤ 0.05; ** *P* ≤ 0.01.

Notes: The sample sizes are shown as number of venues in the major/smaller cities.

**Fig. 2:**
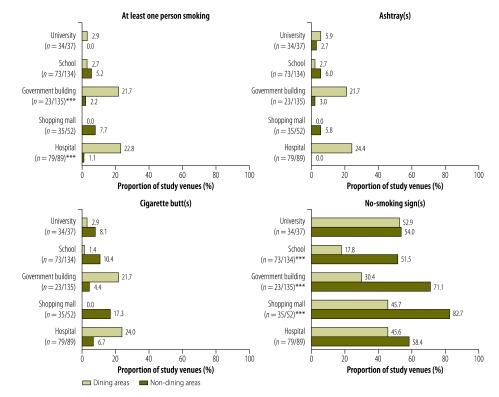
Indoor observations of smoking, ashtrays, cigarette butts and no-smoking signs in the dining and non-dining areas of public venues, Turkey, 2012–2013

In the non-hospitality venues that had both dining and non-dining areas, smoking was observed either more or less often in the dining area than in the non-dining areas – depending on venue type (Fig. 2). In both government buildings (21.7% versus 2.2%; *P* < 0.001) and hospitals (22.8% versus 1.1%; *P* < 0.001), for example, smoking was observed in a much greater proportion of the dining areas than of the non-dining areas. Among the indoor non-dining areas of schools, smoking was observed in two main entrances, two offices, two toilet areas and a fire escape. Within the shopping malls, smoking was observed in five non-dining locations: a main entrance, a hallway/walkway, a toilet area, a fire escape and a tailor’s shop.

*** *P* ≤ 0.001.

Notes: The sample sizes are shown as number of dining/non-dining areas observed.

**Fig. 3:**
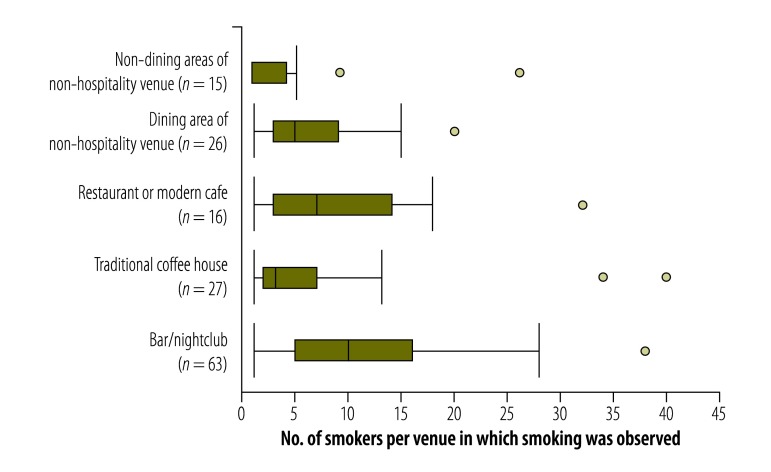
Numbers of smokers observed within venues where any smoking was observed, Turkey, 2012–2013

Notes: In each of the box-and-whisker plots, the line within the box indicates the median, the box indicates the interquartile range, the error bars indicate one and a half times the length of the box from either end of the box, and the circles indicate outlying data points.

Smoking was observed in just four (6.0%) of the 67 cafes but in 63 (79.7%) of the 79 bars/nightclubs (Table 1). Among the venues in which any smoking was observed, the bars/nightclubs gave the highest median number of observed smokers per venue (Fig. 3).

Ashtrays were seen in about one of every five dining areas in government buildings and hospitals (Fig. 2), about one of every five traditional coffee houses, and about four of every five bars/nightclubs (Table 1). They appeared to be relatively rare in other study locations and venues. In general, the presence of cigarette butts mirrored that of smoking and ashtrays, although cigarette butts were observed more often than smoking or ashtrays (Table 1, Fig. 1 and Fig. 2). The proportions of indoor locations in which at least one ashtray or cigarette butt was observed were positively correlated with the number of smokers observed in that type of location (*r* = 0.85 for ashtrays and 0.82 for cigarette butts; further information available from the corresponding author). In bars/nightclubs, the PM_2.5_ concentrations in indoor air were found to be moderately correlated with the number of smokers observed (*r* = 0.32; further information available from the corresponding author).

The proportions of venues in which indoor no-smoking signs were observed ranged from 54.5% (73/134) for schools to 85.8% (103/120) for coffee houses (Table 1), with no major differences in the values for large and small cities (Fig. 1). In government buildings, malls and schools, such signs were significantly less likely to have been observed in dining areas than in non-dining areas (*P* < 0.001; Fig. 2). In most venues, the observed no-smoking signs were considered to be obvious, with no differences by city size (available from the corresponding author). Most of the observed signs included details of the fines for smoking (862/1032).

After adjustment for any ashtrays, signs and cigarette sales, the proportions of traditional coffee houses and bars/nightclubs in which smoking and cigarette butts were observed were still higher than the corresponding values for the non-hospitality study venues – although the apparent strength of these associations was weakened by the adjustment (Table 2). The presence of ashtrays was associated with the presence of smoking and cigarette butts, both before and after adjustment for the other variables. After adjustment, the presence of no-smoking signs was associated with a reduction in the likelihood that smoking (aOR: 0.8; 95% CI: 0.4–1.5) or cigarette butts (aOR: 0.5; 95% CI: 0.3–0.8) would be observed in a venue – although the association was significant only for cigarette butts. After adjustment, cigarette sales – in or close to a venue – were found to be associated with the presence of cigarette butts indoors (aOR: 2.6; 95% CI: 1.1–5.9).

**Table 2:** Associations between the presence of smoking and presence of cigarette butts in indoor public places in 12 cities, Turkey, 2012–2013

Variable	No. of venues	No. of locations	Smoking^a^			Cigarette butt(s)^a^	
			cOR (95% CI)	aOR (95% CI)^b^		cOR (95% CI)	aOR (95% CI)^b^
**Location type**							
Non-hospitality venue^c^							
Non-dining area^d^	447	2422	1.0	1.0		1.0	1.0
Dining area	244	246	17.7 (9.0–34.6)	5.1 (2.0–13.1)		6.7 (3.9–11.3)	1.8 (0.8–3.9)
Restaurant or modern cafe	238	547	6.5 (3.3–12.9)	4.0 (1.6–9.7)		2.5 (1.5–4.4)	1.5 (0.8–3.0)
Traditional coffee house	120	180	26.5 (13.5–52.1)	14.9 (5.6–39.9)		9.5 (5.5–16.4)	4.6 (2.1–10.1)
Bar or nightclub	79	266	108.7 (60.2–196.3)	12.1 (5.4–27.3)		47.8 (31.1–73.6)	8.3 (4.5–15.1)
**Ashtray**							
Not observed^d^	982	3447	1.0	1.0		1.0	
Observed	145	211	608.1 (352.9–1047.7)	315.9 (174.9–570.8)		267.8 (170.8–420.0)	165.4 (98.0–279.1)
**No-smoking sign**							
Not observed^d^	435	2629	1.0	1.0		1.0	1.0
Observed	693	1032	1.9 (1.5–2.5)	0.8 (0.4–1.5)		1.4 (1.1–1.9)	0.5 (0.3–0.8)
**No-smoking sign/ashtray**							
Observed/not observed^d^	603	938	1.0	1.0		1.0	1.0
Not observed/not observed	379	2509	0.9 (0.4–2.0)	1.3 (0.5–3.1)		2.1 (1.1–4.3)	2.6 (1.3–5.4)
Observed/observed	89	93	611.8 (250.0–1496.9)	342.3 (129.5–904.7)		439.1 (190.2–1013.9)	232.6 (95.7–564.4)
Not observed/observed	56	118	512.1 (221.0–1186.6)	396.5 (155.0–1014.4)		521.2 (229.9–1181.5)	353.9 (147.1–851.8)
**Cigarette sales**							
Not observed^d^	1026	ND^e^	1.0	1.0		1.0	1.0
Observed	102	ND^e^	0.8 (0.4–1.5)	1.5 (0.6–4.0)		0.8 (0.5–1.6)	1.9 (0.9–3.9)

aOR: adjusted odds ratio; CI: confidence interval; cOR: crude odds ratio; ND: not determined.

^a^ Odds ratios were estimated in logistic regression models, with generalized estimating equations used – for all of the variables evaluated except for cigarette sales because this variable was only recorded at venue level – to account for the clustering of study locations within study venues.

^b^ Adjusted models included all the other variables shown in the table.

^c^ Government buildings, hospitals, schools, shopping malls and universities.

^d^ Used as a reference category.

^e^ Cigarette sales were only recorded at venue level and not at location level.

### Outdoor locations

**Fig. 4:**
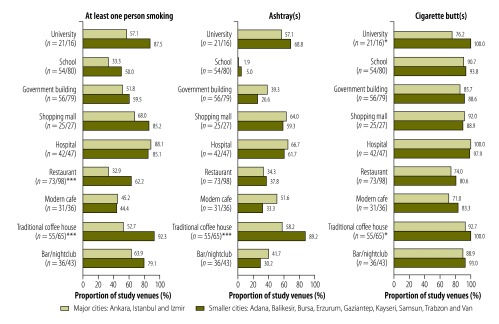
Outdoor observations of smoking, ashtrays and cigarette butts in 12 cities, Turkey, 2012–2013

In general, fieldworkers were more likely to see people smoking in the outdoor locations they investigated than in the indoor locations at the same venues (Table 1). Smoking in the outdoor areas of coffee houses and restaurants was less often observed in the cities of Ankara, Istanbul and Izmir than in the smaller cities, 92.3% (60/65) versus 52.7% (29/55; *P* < 0.001) and 62.2% (61/98) versus 32.9% (24/73; *P* < 0.001), respectively (Fig. 4). 

* *P* ≤ 0.05; *** *P* ≤ 0.001.

Notes: The sample sizes are shown as number of venues in the major/smaller cities.

The number of outdoor locations (in major and smaller cities) in which cigarette butts were observed was very high, ranging from 77.6% (52/67) around cafes to 98.9% (88/89) around hospitals. Outdoor cigarette butts were found predominantly on the ground.

The correlations between the numbers of smokers and ashtrays (*r* = 0.49) and smokers and cigarette butts (*r* = 0.37) observed in outdoor locations were moderate (further information available from the corresponding author). The PM_2.5_ concentrations in the outdoor air near the main entrances and on the patios and terraces of bars/nightclubs were moderately positively correlated with the number of smokers observed in the same locations (*r* = 0.55). After adjustment, bars/nightclubs, presence of ashtrays and presence of cigarette sales were found to be associated with the observation of outdoor smoking, and ashtrays and cigarette sales were found to be associated with the observation of cigarette butts outdoors (Table 3).

**Table 3:** Associations between the presence of smoking and presence of cigarette butts in outdoor areas around public venues in 12 cities, Turkey, 2012–2013

Variable	No. of venues	No. of locations	Smoking^a^			Cigarette butt(s)^a^	
			cOR (95% CI)	aOR (95% CI)^b^		cOR (95% CI)	aOR (95% CI)^b^
**Location type**							
Non-hospitality venue^c^							
Non-dining area^d^	447	739	1.0	1.0		1.0	1.0
Dining area	23	23	22.5 (3.0–168)	5.7 (0.7–44.5)		1.7 (0.5–5.8)	0.8 (0.2–2.9)
Restaurant or modern cafe	238	326	0.7 (0.6–1.0)	0.7 (0.5–0.9)		0.6 (0.4–0.8)	0.5 (0.4–0.7)
Traditional coffee house	120	164	2.2 (1.5–3.2)	1.3 (0.9–2.0)		1.8 (1.1–2.9)	1.3 (0.8–2.2)
Bar or nightclub	79	104	2.2 (1.4–3.5)	2.4 (1.5–4.0)		2.4 (1.2–4.7)	2.3 (1.2–4.6)
**Ashtray**							
Not observed^d^	521	886	1.0	1.0		1.0	1.0
Observed	386	469	6.5 (5.0–8.4)	6.0 (4.6–7.9)		2.8 (2.1–3.9)	2.9 (2.0–4.0)
**Cigarette sales**							
Not observed^d^	826	ND^e^	1.0	1.0		1.0	1.0
Observed	81	ND^e^	7.4 (3.4–16.3)	4.7 (2.0–10.9)		5.6 (1.4–23.2)	4.8 (1.1–21.6)

aOR: adjusted odds ratio; CI: confidence interval; cOR: crude odds ratio; ND: not determined.

^a^ Odds ratios were estimated in logistic regression models, with generalized estimating equations used – for all of the variables evaluated except for cigarette sales because this variable was only recorded at venue level – to account for the clustering of study locations within study venues.

^b^ Adjusted models included all the other variables shown in the table.

^c^ Government buildings, hospitals, schools, shopping malls and universities.

^d^ Used as a reference category.

^e^ Cigarette sales were only recorded at venue level and not at location level.

## Discussion

In this evaluation of compliance with smoke-free legislation across 12 cities in Turkey, we found good compliance in the non-dining areas of government buildings, hospitals and universities – since smoking was observed in 2% or less of such areas. Smoking was also observed in less than 10% of the non-dining areas studied in cafes, malls, restaurants and schools. However, compliance appeared to be poor in coffee houses and the dining areas of government buildings and hospitals and very poor in bars/nightclubs. Smoking appeared to be especially common in the outdoor locations close to bars/nightclubs, coffee houses, hospitals, malls and universities.

In Turkey, hospitality venues were given a period of 18 months to adopt the new smoke-free legislation.^7^ Although similar adoption periods for hospitality venues were used by Belgium,^17^ Chile^18^ and Spain^19^ when they introduced smoke-free legislation, countries such as Ireland^20^ and Uruguay^21^ implemented their smoke-free legislation simultaneously and successfully in all of their public venues. It is impossible to know whether implementing the law for all public places simultaneously in Turkey would have been more successful – but staggering the introduction of smoke-free legislation can add confusion which complicates implementation and enforcement.^10^

Our results indicate that outdoor and – especially – indoor ashtrays could be major facilitators of smoking in urban Turkey. The presence of an ashtray in an area where smoking is prohibited provides a conflicting message. In a study of 75 hospitality venues in five cities in Greece, PM_2.5_ concentrations were strongly associated with the presence of ashtrays.^22^ Ashtrays are modifiable determinants of smoking behaviour and should be removed from all indoor public places. Our data indicated that the presence of no-smoking signs reduced the likelihood of cigarette butts being observed in the same locations. Such signs, however, were observed in less than 70% of the bars/nightclubs, cafes and dining areas in government buildings and hospitals that we investigated. After adjustment, cigarette sales – another possible facilitator of smoking behaviour^23^ – were associated with cigarette butts both indoors and outdoors and with smoking in outdoor areas.

The general lack of compliance seen in the hospitality venues we studied is consistent with the high PM_2.5_ concentrations recorded indoors in other studies in Turkey that used convenience sampling and were limited to hospitality venues.^8^^,^^10^ Our findings are also consistent with those reported for Turkey by the Global Adult Tobacco Survey – e.g. that exposure to second-hand smoke occurred in 6.0% of health-care facilities, 11.3% of government buildings and 55.9% of restaurants in 2008^24^ and that the corresponding values for 2012 were 3.8%, 6.5% and 12.9%, respectively.^6^ The same survey reported that, between 2008 and 2012, the percentage of adults visiting cafes, coffee houses or tea houses who reported exposure to second-hand smoke in these venues fell from 55.9% to 26.6%.^6^^,^^24^ However, the Global Adult Tobacco Survey has not included specific questions about areas with particular challenges for implementation, such as bars/nightclubs and the dining areas of government buildings and hospitals. Our results therefore include information that is complementary to the data recorded by the Global Adult Tobacco Survey. Three other surveys related to the smoke-free legislation introduced in Turkey in 2008 have been relatively small-scale and have focused on opinions on the smoking ban rather than on the ban’s enforcement.^25^^–^^27^

In several other countries, as in Turkey, compliance with smoke-free legislation has been found to be lower in hospitality venues than in other public places. In India, for example, 65% of the educational institutions and health-care facilities were found to be free of people smoking compared to 37% of the eateries.^28^ In Guatemala, following the enactment of smoke-free legislation in 2009, air nicotine concentrations were found to be higher in bars and nightclubs than in other public places.^29^ Although the dining areas in Turkey’s government buildings and hospitals are generally run by private catering companies, they remain under the jurisdiction of the host institutions and the institutions’ directors should be accountable for compliance. The enforcement of the smoke-free legislation could be made a condition of any catering subcontracts.

We used a guide on compliance studies^12^ to evaluate the implementation of Turkey’s smoke-free legislation on a large scale. While the guide has been used previously, few studies have implemented it rigorously and comprehensively. In northern India, the guide was used to estimate overall compliance of 23% in a tertiary hospital^30^ and 92% in educational institutions, government offices, health-care facilities, hospitality venues, hotels, shopping malls and transit stations.^31^

Some of the strengths of our study include the use of a systematic protocol and training and the random sampling strategy followed in each city. As fieldworkers were unable to observe areas of the studied government buildings, hospitals and universities that are inaccessible to the public, levels of compliance in these areas remain unknown. Bars/nightclubs were generally evaluated in the evening whereas coffee houses were generally evaluated in the afternoon. Compliance in the coffee houses during the evening may also have been poor. We found no major differences between the major cities that we studied and the smaller cities. However, the major cities were evaluated in the winter – when more people spend time inside and indoor compliance could be worse than in the summer. We are unable to determine if our results are representative of other cities, towns and communities in Turkey or whether compliance in rural areas of Turkey is similar to that which we recorded.

Widespread smoking behaviour contributes to maintaining the social acceptability of smoking.^32^ Our observational data from Turkey are relevant for public health professionals and entities responsible for protecting the public from exposure to second-hand smoke. During a dissemination meeting, we distributed the city-specific results of our study to inspectors and civil servants from the Ministry of Health of Turkey who work in each of our study cities. Our results indicate possible actions by the Ministry of Health, other responsible agencies, public health professionals and venue directors and managers, such as the elimination of ashtrays, the wider distribution of no-smoking signs and the tighter regulation of cigarette sales in public places. In outdoor areas, near entrances and on patios/gardens, exposure to second-hand smoke is widespread and our findings support the need for additional legislation to protect individuals who spend time in such areas. 
